# Computational NMR Study of *Cryptolepis* Alkaloids: Could the Structural Misassignment of Cryptospirolepine Have Been Avoided?

**DOI:** 10.3390/ijms26052112

**Published:** 2025-02-27

**Authors:** Valentin A. Semenov, Leonid B. Krivdin, Gary E. Martin

**Affiliations:** 1 A.E. Favorsky Irkutsk Institute of Chemistry, Siberian Branch of the Russian Academy of Sciences, Favorsky St. 1, 664033 Irkutsk, Russiakrivdin55@gmail.com (L.B.K.); 2 Department of Chemistry and Biochemistry, Seton Hall University, 400 South Orange Ave., South Orange, NJ 07079, USA

**Keywords:** *Cryptolepis* alkaloids, cryptospirolepine, ^13^C NMR, DFT calculations

## Abstract

It is demonstrated that by using modern computational methods, it is possible to identify potential structural misassignments for even molecules as complex as cryptospirolepine. While methods that rely on anisotropic nuclear magnetic resonance (NMR) methods can also be used to offer a powerful orthogonal means of structure elucidation and verification, the present computational study should be feasible in most chemistry departments with access to sophisticated computational facilities. From the overall correlations calculated against experimental ^1^H and ^13^C NMR chemical shifts in the series of thirteen *Cryptolepis* alkaloids, it follows that, generally, they provide a very high level of correlation. The corrected mean absolute error (CMAE) is found to be at the level of 0.13 and 1.5 ppm for, proton and carbon chemical shifts, respectively, which is only about 2 and 1% of their ranges. Based on the performed calculations, some spectral reassignments are suggested, and a number of missing (unknown) experimental chemical shifts are predicted.

## 1. Introduction

**Figure 1:**
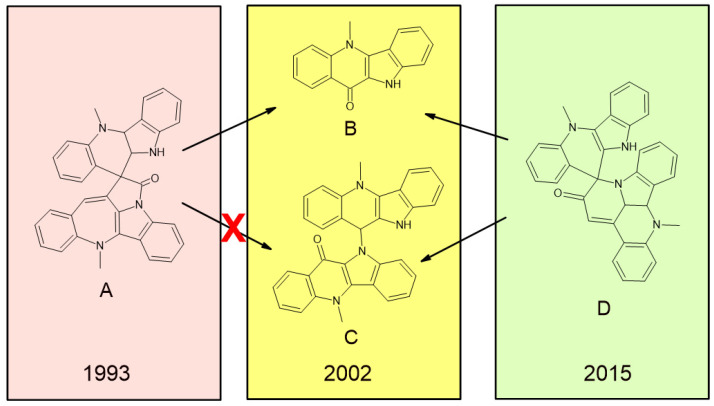
The original structure of cryptospirolepine, A, reported in 1993 [3], did not allow the structure of the degradants, B and C, isolated from a degraded NMR sample in 2002 [4], to be rationalized. It took more than a decade from the 2002 report of the structure of the degradants for technology to advance far enough to allow the minimal amount of remaining cryptospirolepine to be fully characterized as D using the, then newly developed, 1,1-HD-ADEQUATE experiment [5]. The structure has since been confirmed independently using both anisotropic NMR methods [6] and the very recently developed i-HMBC technique that allows the unequivocal differentiation of two- from three-bond heteronuclear correlations on the basis of sub-ppb isotope shifts [7]. Reproduced with minor editing privilege from [6].

Numerous alkaloids have been isolated and characterized from the West African medicinal plant *Cryptolepis sanguinolenta*. The first member of the family of alkaloids to be isolated was the indoloquinoine alkaloid cryptolepione [1,2]. The most complex and interesting member of the series is the spirononacycllic alkaloid cryptospirolepine, labeled A in Figure 1, whose structure was first reported in 1993 [3]. However, it was not until a degraded NMR sample of the alkaloid from a sealed NMR tube was examined chromatographically with the two major components isolated and fully characterized, nearly a decade after the first report, that it became obvious that the structure of cryptospirolepine had, indeed, been misassigned [4]. It was evident that one of the two major degradants, labeled C, could not be mechanistically or structurally rationalized from the originally reported structure of A, as shown in Figure 1. The structure of cryptospirolepine was not corrected to the structure labeled D in Figure 1 until a, then newly reported, 1,1-HD-ADEQUATE spectrum was acquired for a submilligram sample of the alkaloid using a 600 MHz spectrometer equipped with a 1.7 mm MicroCryoProbe™ in a 120 h long experiment in a 2015 study [5].

**Scheme 1:**
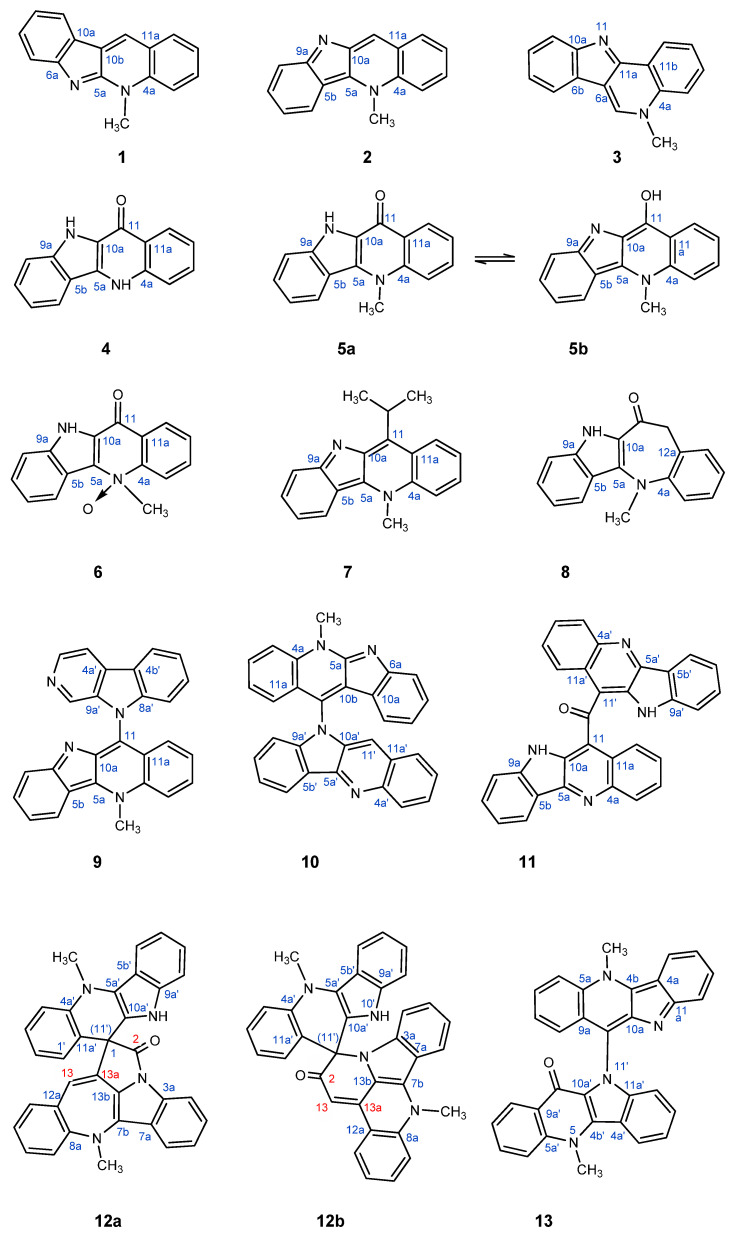
*Cryptolepis* alkaloids investigated computationally in the present study. Structures **5a** and **5b** are in tautomeric equilibrium; the originally reported incorrect structure of cryptospirolepine is shown as **12a**, while the revised structure is shown as **12b**. The carbons in the two structures whose positions in the molecular framework have changed are labeled in red. The spiro carbons in both **12a** and **12b** are labeled as C-1/(C-11′) for clarity. Finally, a degradation product of cryptospirolepine [4], which is not a naturally occurring *cryptolepis* alkaloid, is shown (**13**).

Obviously, accurately calculating the ^13^C NMR chemical shifts in a molecule as complex as cryptospirolepine was well beyond computational capabilities when the structure was first reported in 1993 [3]. The evolution of NMR probe technology and newly developed NMR methods finally, in 2015, allowed the correct structure of cryptospirolepine (D) to be determined (Scheme 1). The structure reported in 2015 has since been unequivocally confirmed through the application of anisotropic NMR techniques in a 2017 report [6], and again more recently using the recently developed i-HMBC technique that allows the unequivocal differentiation of two-bond from three-bond and longer-range heteronuclear correlations on the basis of sub-ppb isotope shifts [7].

Over the more than 70 years since the initial isolation of quindolinone, a number of other members of this intriguing family of indoloquinoline alkaloids have been isolated and characterized. These have included: cryptotackieine (**1**), a linear indolo[2,3-*b*]quinoline [8]; cryptolepine (**2**), an indolo[3,2-*b*]quinoline [1,2,9]; cryptosanguinolentine (**3**), an angular isomeric indolo[3,2-*c*]quinoline [8]; quindolinone (**4**) [10]; oxidation at the 11-position of the cryptolepine skeleton has been variously reported for afford cryptolepinone (**5a**) or 11-hydroxycryptolepine (**5b**) [11,12,13]; a facile *N*-oxidation product of cryptolepinone (**6**) has also been reported [14] as has an 11-*i*-propyl analog (**7**) [15]. While most members of the *Cryptolepis* alkaloid family have involved indoloquinoline frameworks, one indolobenzazepine analog, homocryptolepinone (**8**), has been reported [16]. More complex examples include cryptolepicarboline (**9**) [17]; quindolinocryptotackiine (**10**) [18]; the bis-indoloquinoline cryptomisrine (**11**) [19]; and the initial incorrect (**12a**) and subsequently corrected (**12b**) structures of cryptospirolepine [3,5]. Finally, a complex degradation product of cryptospirolepine, 5.5′-*dimethyl*-5′*H*-10,11′-biindolo[*3,2-b*]quinoline-11(5*H*)one, which is essentially an indoloquinoline dimer, has also been isolated and characterized (**13**) [4].

## 2. Results and Discussion

The initial structural misassignment of cryptospirolepine as **12a [3],** based on the then available NMR methods and probe technology, which was subsequently corrected to **12b** in 2015 [5] (see also Figure 1), coupled with the confusion over whether the structure of **5a** or **5b** was correct based on the available NMR data, prompted us to embark on the present computational study. There also exists the possibility that an as-yet-undiscovered indolo[2,3-*c*]quinoline analog of cryptosanguinolentine (**3**, indolo-[3,2-*c*]quinoline) may be discovered that could benefit from an in-depth analysis of the proton and carbon chemical shift behavior of existing *Cryptolepis* alkaloids. In part, we also undertook this study based on the growing numbers of natural product structural reassignments and corrections appearing in the literature.

Recent developments in anisotropic NMR methods, as highlighted in a recent chapter [20] and several pivotal earlier reports [6,21], provide a powerful orthogonal means of confirming or refuting structural proposals, but require undertaking additional experimental studies that may be beyond the abilities of a given investigator’s lab. In contrast, computational methods, such as those embodied in the present study, can be confidently undertaken in most modern chemistry departments, making them a potential alternative to anisotropic NMR studies.

Therefore, in the course of theoretical part of this study, the three-dimensional structures of the natural alkaloids under scrutiny were primarily established. To achieve this goal, a preliminary conformational search was carried out for each of the structures studied. In this case, for compounds **9**–**13**, the existing experimental NOE correlations between the two alkaloid “subunits” were taken into account. In this way, the conformational search was reduced mainly to the detection of exact rotational angles around the subunit linkages. As a result, in the process of a conformational search in the range of about 0–10 kcal/mol, several groups of the low-energy conformers were identified for each of the compounds **1**–**13**.

Next, the geometric parameters of the conformers found were optimized at a higher density functional theory (DFT) level, namely M06-2X/cc-pVTZ//aug-cc-pVTZ. A cc-pVTZ basis set was assigned to the carbon and hydrogen atoms, and aug-cc-pVTZ to the nitrogen and oxygen atoms, the latter for a more accurate definition of the diffuse region in the field of their LEPs. In the next step, for the accurate geometries obtained, the ^1^H and ^13^C shielding constants were calculated and then converted into the corresponding NMR chemical shifts, see the Section 3. Using an array of experimental spectral data and theoretical calculation data, a comprehensive correlation assessment of all considered stereoisomers was carried out, taking into account such statistical descriptors as CMAE, root mean square deviation (RMSD) and Pearson correlation coefficient.

**Figure 2:**
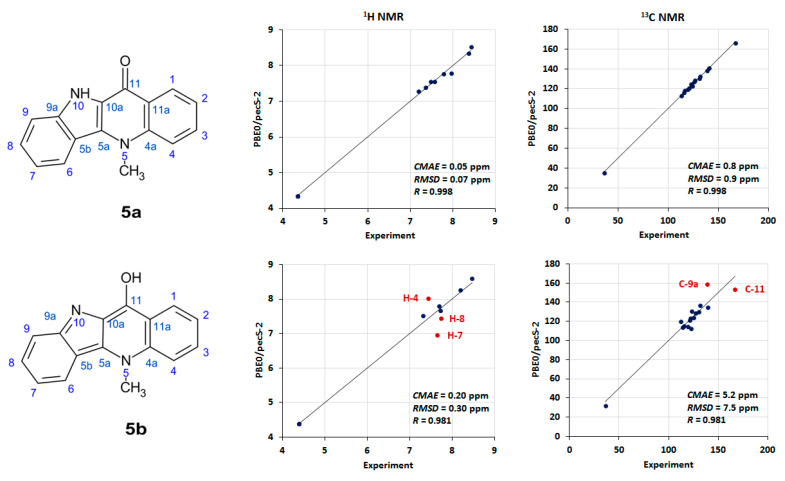
NMR correlation analysis for the structural assignment of tautomeric equilibrium of cryptolepinone (**5a**) (**top**) and 11-hydroxycryptolepine (**5b**) (**bottom**). Selected points with significant deviations are shown in red. Here and below (Figures 4 and 5) on the correlation plots: the abscissa axis presents the experimentally observed values of NMR chemical shifts, while the ordinate axis reveals their theoretically calculated counterparts.

Compound **5a**/**5b** was used as a starting point because it is a convenient model for developing a methodology for the further (and most interesting) computational study of cryptospirolepine **12a**/**12b**. Therefore, the first point of discussion pertains to the interpretation of the NMR data acquired for **5a**/**5b**, not to the validity of the overall structure of the molecule. In the initial report by one of the authors, the structure was assigned as the indoloquinone form, **5a**, based on the acquired NMR data [10]. A subsequent synthetic report by Jolie and co-workers [11] reported the structure as 11-hydroxy cryptolepine, **5b**. When examining the proton and carbon NMR data for both structures in Figure 2, it is quite apparent that there is significantly better agreement between the calculated and observed ^1^H and ^13^C chemical shift data for the quinolinone structure, **5a**, than for the 11-hydoxycyptolepine, **5b**, conformer of the molecule. When the graphical plots of the data presented in Figure 2 are considered, the scatter in the proton data for the H-4, H-8, and H-7 resonances for the 11-hydoxy form is quite apparent. The error is also pronounced for the C-9a and C-11 carbon chemical shifts in the 11-hydroxy form (**5b**), whereas there is excellent congruence between the ^1^H and ^13^C chemical shift data for all of the resonance of quindolinone, **5a**. For the ^13^C chemical shift in the C9a resonance, this is not particularly surprising given that there is a C-9a=N-10 bond in the cryptolepine form of the molecule, **5b**, that tends to shift that carbon resonance considerably downfield of where it would be observed as the C-9a–N-10 single bond in quindolinone, **5a**. In this case, if a computational study such as the present one had been possible when those compounds were synthesized, the misassignment of the isomeric form of the molecule could have been avoided.

The picture presented by the assignment of the structure of cryptospirolepine, **12a**, is considerably more complex [3], as shown in Scheme 1. When the original significantly discolored sealed NMR tube containing the sample was cut open and examined chromatographically in 2002 [4], it became apparent from the structural characterization of one of the two major degradants contained in the sample (there were a total of sixteen peaks observed in the HPLC chromatogram of the sample when injected prior to isolation efforts) that the degradant could not have arisen from the structure initially reported for complex alkaloid in 1993 [3]. One of the major degradants isolated and characterized was cryptolepinone, whose structure can, by contrast, be mechanistically derived from the originally proposed structure of cryptospirolepine (**12a**) [4]. The second major degradant was characterized by NMR spectroscopic methods, in conjunction with a computer-assisted structure elucidation (CASE) study as 5,5′-dimethyl-5′*H*-10,11-biindolo[3,2*-b*]quinoline-11(5*H*)-one (**13**) [4], which cannot be mechanistically rationalized by the originally reported structure of cryptospirolepine (**12a**).

It took more than a decade and the development of a 600 MHz 1.7 mm MicroCryoProbe™ gradient triple resonance probe in conjunction with the development of homodecoupled (HD) variants of the 1,1- and 1,n-ADEQUATE experiment [4] to be able to correctly characterize the remaining 700 μg sample of the alkaloid in an experiment that consumed 120 h of spectrometer time using a 1.7 mm MicroCryoProbe™-equipped 600 MHz spectrometer, finally establishing the correct structure of the alkaloid as **12b**, in which the benzazepine ring was revised to a pyridinone moiety. The structure of **12b** has subsequently been orthogonally confirmed using anisotropic NMR methods [6] and the newly developed i-HMBC experiment that can unequivocally differentiate ^2^*J*_CH_ from ^3^*J*_CH_ correlations on the basis of isotope shift differences [7].

**Figure 3:**
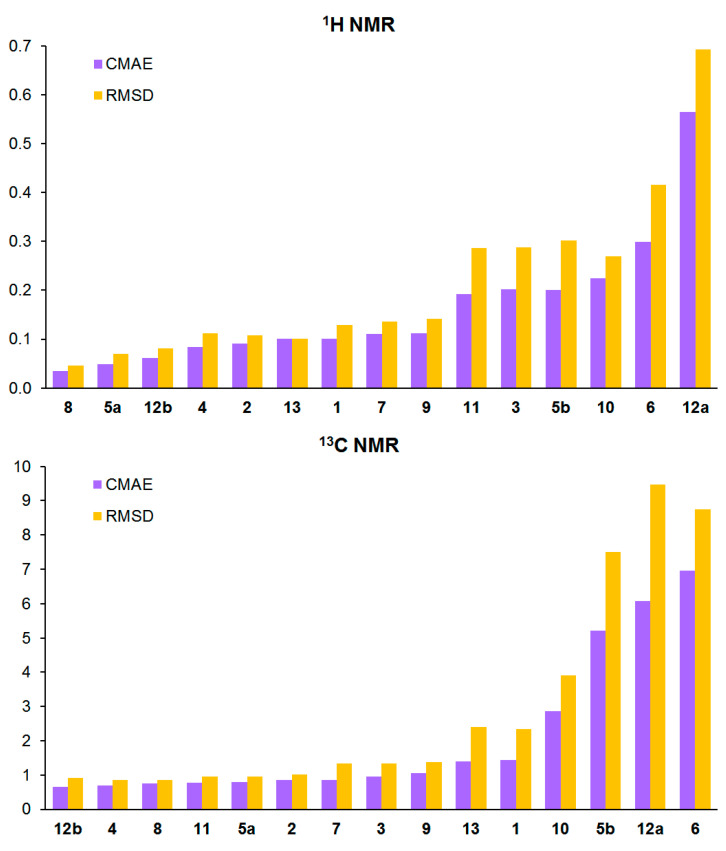
Corrected mean absolute error (ppm) and root-mean-square deviation (ppm) of calculated ^1^H and ^13^C NMR chemical shifts in *Cryptolepis* alkaloids **1**–**13**. The graphs are arranged in the order of increasing their CMAE values.

Figure 3 presents the CMAE and RMSD bar charts for the calculated ^1^H and ^13^C NMR chemical shifts in compounds **1**–**13**. Each individual bar for each compound was obtained by correlating the theoretical and experimental chemical shifts using Equations (2) and (3), see the Section 3. As is readily apparent from the graphical bar data in Figure 3, while the revised structure of cryptospirolepine (**12b**) gave excellent congruence between the calculated and observed ^1^H and ^13^C chemical shift data, in contrast, the originally reported incorrect structure, **12a**, essentially had the worst correlation data of any of the molecules considered in this study.

**Figure 4:**
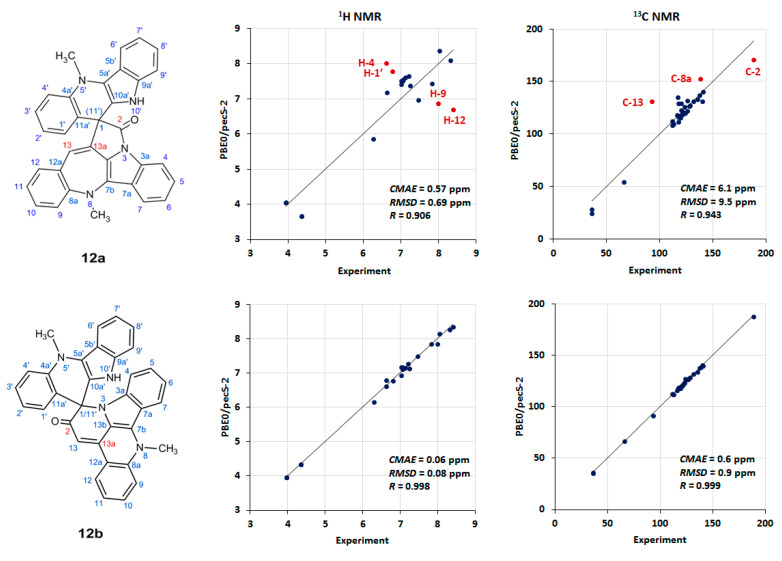
NMR correlation analysis for structural assignment of cryptospirolepine (**12a**/**12b**). Top: plots of the calculated vs. experimental ^1^H and ^13^C NMR data for the incorrect structure of cryptospirolepine (**12a**) reported in 1993 [3]. Bottom: plots of the calculated *vs*. experimental ^1^H and ^13^C NMR data for the revised structure of cryptospirolepine (**12b**) reported in 2015 [5]. Selected points with significant deviations are shown in red.

Referring to Figure 4, it can be readily seen that the C-2, C-8, and C-13 carbon resonances of the azepine ring were significant outliers in the plotted data. Had the present computational study been possible when the alkaloid was originally isolated and characterized, these data would have signaled a problem with the proposed structure that would have, in turn, prompted further experimental investigation.

In an unrelated study using anisotropic NMR methods reported in 2017 in *Science* [6], one of the authors demonstrated how the indoloquinoline-derived portion of the cryptospirolepine structure was consistent with the reported structure in terms of both the residual dipolar coupling (RDC) and residual chemical shift anisotropy (RCSA), while, in contrast, the indolobenzazepine-derived portion of the molecule in the originally proposed structure (**12a**) exhibited severe aberration between the calculated and observed data. Once again, had the present computational study been conducted on the original data, it would have been evident that there was a structural misassignment that could have been further investigated that might have averted the mistaken original report of the structure. While the anisotropic NMR study does, indeed, afford a method for the orthogonal assessment of the validity of complex natural product structures, such NMR studies may be beyond the scope of what is possible in some investigators’ laboratories. In contrast, the more recently reported i-HMBC method [7] is no more experimentally challenging to perform than a conventional HMBC experiment, and could also have avoided the structural misassignment.

As for the other *Cryptolepis* alkaloids studied, it should be noted that, generally, they provide a high level of correlation between theoretically calculated and experimental values of ^1^H and ^13^C NMR chemical shifts, see Figure 3. The average CMAE is 0.05–0.20 ppm for ^1^H and 0.5–3.0 ppm for ^13^C NMR chemical shifts. However, in addition to the described particular features of **5b** and **12a**, the behavior of cryptolepinone 5(*N*)-oxide (**6**) is particularly noteworthy. It is obvious that the (*N*)-oxide-pyridinone moiety is extremely complex for being described in terms of its stereoelectronic structure. A significant spin density is concentrated on the nitrone nitrogen atom, which is partially donated from oxygen. The excess spin density is then distributed throughout the aromatic system of the rest of the molecule. These electronic effects are extremely difficult to accurately describe within the DFT framework. As a result, significant deviations of calculated NMR chemical shifts from the experiment arise, and this is especially so for carbon nuclei. For a more detailed distribution of these deviations, see Supporting Information—Figure S1.

**Scheme 2:**
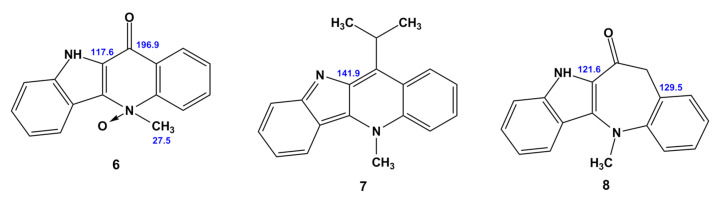
Missing ^13^C NMR data for **6**–**8**; computational assignments are based on the performed calculations.

As a result of the performed calculations for the whole series of **1–13**, several missing (unknown) experimental ^13^C NMR chemical shifts were predicted with a high level of confidence, which are provided in Scheme 2.

**Scheme 3:**
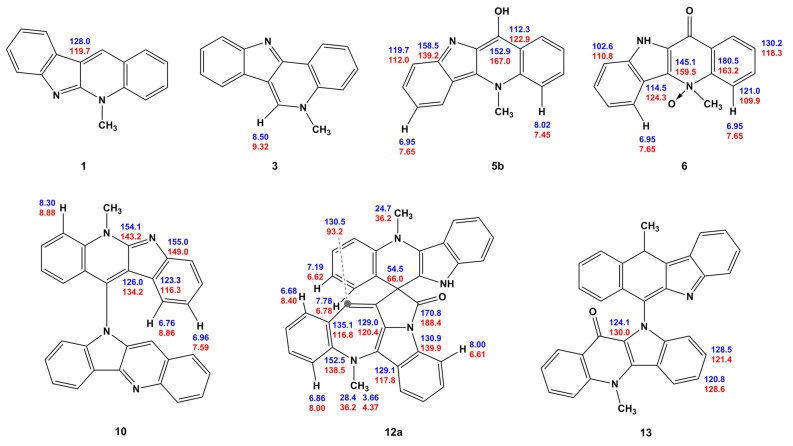
Selected ambiguous values of NMR chemical shifts (ppm) in *Cryptolepis* alkaloids **1**, **3**, **5b**, **6**, **10**, **12a**, **13**. Red—experimental values, blue—computational assignments based on the performed calculations.

Besides of the mentioned deviations in **5a**/**5b**, **6** and **12a**/**12b**, there are also a number of established deviations in other compounds, see Scheme 3. Most of them are related to the underestimation of the electron correlation effects at the applied level of approximation, GIAO-DFT/[triple-zeta basis set]. However, based on the performed calculations, a number of reassignments are most likely justified, as exemplified by C-9 and C-11a in **5b**.

**Figure 5:**
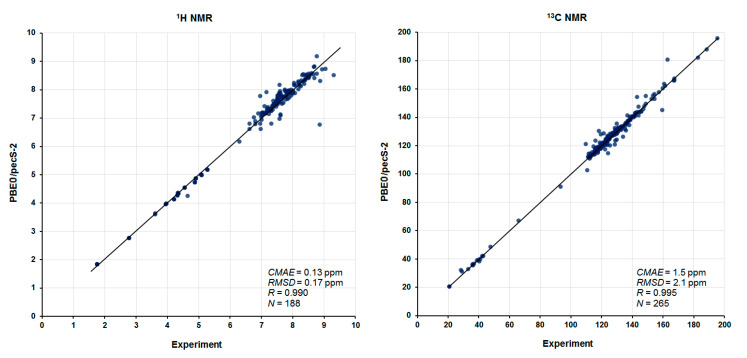
Overall correlation plots of calculated vs. experimental ^1^H and ^13^C NMR chemical shifts of **1**–**13**, ppm.

As a final illustration, Figure 5 shows the overall correlation plots calculated against experimental ^1^H and ^13^C NMR chemical shifts in the complete series of alkaloids, **1**–**13**. A total number of 188 ^1^H and 265 ^13^C NMR chemical shifts were analyzed in this study, which provides a statistically significant set for correlation analysis. As follows from the correlations presented, the CMAE is at the level of 0.13 and 1.5 ppm, respectively, for the ^1^H and ^13^C NMR chemical shifts, which is only about 2 and 1%, respectively, of their studied chemical shift ranges. At that, the RMSD is only 0.17 and 2.1 ppm for ^1^H and ^13^C NMR chemical shifts, respectively.

For more clarity, we present Table 1 and Table 2, providing calculated and experimental ^1^H and ^13^C NMR chemical shifts in compounds **9**–**13**, each containing two alkaloid subunits. These data demonstrate how the variation in chemical environment reflects the values of chemical shifts in particular atoms for the most demanding compounds from the studied series. For more detailed analysis, all calculated ^1^H and ^13^C NMR chemical shifts of **1**–**13** are collected in Tables S1 and S2, see Supporting Information.

## 3. Materials and Methods

The initial conformational search of compounds **1**–**13** was performed using the OPLS3 force field in the liquid phase of particular solvent, employing the MacroModel module implemented in the Schrödinger Maestro 11.5 package (Schrödinger, LLC: New York, NY, USA) [22]. During this search, approximately 10^5^ steps were performed to identify the most probable conformers. Subsequently, the unique conformations of the compounds were identified and subjected to further geometry optimization using the GAUSSIAN 09 program (Gaussian, Inc.: Wallingford, CT, USA) [23] at the M06-2X/cc-pVTZ//aug-cc-pVTZ level [24,25]. The solvation effect was taken into account using the IEF-PCM model developed by Tomasi [26,27]. After the optimization process had reached the potential energy surface minima for each of the studied compounds **1**–**13**, their final geometries were retained and used for further calculation of the shielding constants and corresponding NMR chemical shifts.

Calculations of ^1^H and ^13^C NMR isotropic magnetic shielding constants were carried out at the GIAO-DFT level in the liquid phase of particular solvent using the GAUSSIAN 09 program. In these calculations, we employed the one-parameter hybrid functional PBE0 [28], which was used in combination with Rusakov’s basis set, pecS-2 [29,30]. Further, the calculated NMR shielding constants of all significant conformers found by the conformational search were averaged according to the Boltzmann distribution equation:

(1) $$\sigma^{i}=\frac{\sum_{n}\sigma_{n}^{i}e^{\left(-E_{n}/RT\right)}}{\sum_{n}e^{\left(-E_{n}/RT\right)}}$$

where *σ_n_^i^* is the shielding constant of nucleus *i* in conformer *n*, *E_n_* are the Gibbs free energies (J) of each of the significant conformer states *n* (relative to the lowest energy conformer), *R* is the molar gas constant (8.31446 J·K^−1^·mol^−1^), and *T* is the temperature (298 K).

To take systematic errors of calculated chemical shifts into account, we have established correlations between their experimental chemical shifts (*x*) and isotropic magnetic shielding constants (*y*), which were further used to find the linear correlation equations of the *y* = *a*x + *b* type. Thus, the resulting shielding constants *σ^i^* were converted into the corresponding chemical shifts: the parameters *a* and *b* were used for recalculating theoretical shielding constants using the equation *δ_calc_* = (*σ^i^* − *b*)/*a*.

Corrected mean absolute errors (CMAE) were calculated as:

(2) $$CMAE=\frac{\sum_{i=1}^{n}\left|\delta_{exp}-\left(\frac{\sigma^{i}-b}{a}\right)\right|}{n}$$

where *σ^i^* are the unscaled shielding constants for each of the *n* nuclei in the molecule, while *a* and *b* are the slope and intercept of the linear regression *σ^i^* = *aδ_exp_* + *b*.

The root-mean-square deviations (RMSD) were evaluated as:

(3) $$RMSD=\sqrt{\frac{\sum_{i=1}^{n}{\left(\delta_{exp}-\delta_{calc}\right)}^{2}}{n}}$$

where *δ_exp_* and *δ_calc_* are experimental and scaled chemical shifts, respectively, in each of the *n* nuclei.

## 4. Conclusions

We have demonstrated through the model theoretical study that, using modern computational methods, it is possible to identify potential structural misassignments, even for molecules as complex as cryptospirolepine (**12b**). While methods that rely on anisotropic NMR methods, such as RDCs and RCSAs, can also be used [6] to offer a powerful orthogonal means of structure verification, the experimental methodology may not be possible in many laboratories, whereas the present computational study should be feasible in most chemistry departments with access to sophisticated computational facilities.

From the overall correlations calculated against experimental ^1^H and ^13^C NMR chemical shifts in the series of 188 ^1^H and 265 ^13^C NMR chemical shifts of 13 compounds, it follows that, generally, they provide a very high level of correlation. The CMAE is at the level of 0.13 and 1.5 ppm for proton and carbon chemical shifts, respectively, which is only about 2 and 1% of their ranges. At that, the RMSD is only 0.17 and 2.1 ppm for ^1^H and ^13^C NMR chemical shifts, respectively. Based on the performed calculations, some spectral reassignments are suggested, and a number of missing (unknown) experimental chemical shifts are predicted.

## Figures and Tables

**Table 1:** Calculated and experimental ^1^H NMR chemical shifts in compounds **9**–**13**, ppm.

Nucleus	9	10	11	12a	12b	13
1	7.95 (7.60) *^a^*	7.96 (7.86)	8.16 (7.58)			7.47 (7.40)
2	7.63 (7.59)	7.64 (7.79)	7.30 (7.22)			7.59 (7.47)
3	7.99 (7.96)	8.15 (8.23)	7.60 (7.56)			7.15 (7.06)
4	8.55 (8.78)	8.30 (8.88)	8.32 (8.28)	8.00 (6.61)	6.80 (6.63)	8.53 (8.58)
5				7.50 (7.01)	6.94 (7.02)	
6	8.58 (8.60)		8.44 (8.42)	7.55 (7.06)	7.12 (7.06)	8.41 (8.70)
7	7.17 (7.10)	7.87 (7.57)	7.29 (7.37)	8.09 (8.32)	8.28 (8.32)	7.87 (7.92)
8	7.62 (7.52)	7.67 (7.85)	7.50 (7.66)			7.47 (7.53)
9	7.46 (7.44)	6.96 (7.59)	7.08 (7.61)	6.86 (8.00)	7.85 (8.00)	7.88 (7.61)
10		6.76 (8.86)		7.43 (7.83)	7.84 (7.83)	
11				6.97 (7.46)	7.50 (7.47)	
12/12a				6.68 (8.40)	8.34 (8.41)	
13				5.86 (6.27)	6.16 (6.30)	
*N*-Me	4.99 (5.10)	5.17 (5.28)		3.66 (4.37)	4.34 (4.36)	5.01 (5.09)
1′	8.12 (8.26)	7.90 (7.80)		7.78 (6.78)	6.78 (6.81)	7.02 (6.77)
2′		7.77 (7.53)		7.19 (6.62)	6.61 (6.63)	7.44 (7.36)
3′	8.46 (8.49)	7.99 (7.76)		7.64 (7.20)	7.26 (7.21)	7.41 (7.38)
4′	8.20 (8.37)	8.51 (8.31)		7.37 (7.24)	7.13 (7.25)	8.54 (8.66)
5′	8.41 (8.49)					
6′	7.48 (7.40)	8.80 (8.68)		8.36 (8.04)	8.16 (8.05)	7.88 (8.08)
7′	7.54 (7.46)	7.79 (7.52)		7.42 (7.01)	7.17 (7.02)	7.78 (7.80)
8′	7.16 (6.91)	7.85 (7.58)		7.50 (7.05)	7.18 (7.07)	7.31 (7.29)
9′		7.42 (7.11)		7.60 (7.12)	7.16 (7.14)	8.23 (8.05)
11′		7.72 (7.90)				
*N*5′-Me				4.06 (3.94)	3.96 (3.96)	4.54 (4.56)

*^a^* Experimental values are given in parenthesis.

**Table 2:** Calculated and experimental ^13^C NMR chemical shifts in compounds **9**–**13**, ppm.

Nucleus	9	10	11	12a	12b	13
1	125.1 (124.9) *^a^*	128.2 (125.7)	124.5 (122.9)	54.5 (66.0)	66.9 (66.0)	119.9 (120.1)
2	123.7 (123.5)	123.5 (129.2)	127.3 (126.8)	170.8 (188.4)	187.9 (188.6)	132.6 (131.5)
3	128.7 (128.6)	132.8 (133.9)	126.5 (126.2)			116.3 (117.4)
3a				130.9 (139.9)	140.2 (139.9)	
4	115.6 (117.0)	117.9 (119.6)	130.9 (129.8)	112.3 (111.5)	112.2 (111.5)	126.7 (125.8)
4a	132.4 (128.7)	138.0 (138.5)	143.7 (143.2)			115.9 (115.5)
4b						142.2 (142.2)
5				122.3 (126.2)	126.4 (126.2)	
5a	142.8 (142.3)	154.1 (143.2)	147.8 (148.0)			133.1 (133.6)
5b	114.0 (114.9)		121.3 (120.7)			
6	125.5 (125.4)		122.1 (121.5)	122.6 (120.1)	119.8 (120.2)	116.9 (117.9)
6a		155.0 (149.0)				
7	114.8 (117.8)	119.5 (114.9)	120.4 (120.6)	119.2 (123.0)	123.2 (122.9)	129.3 (129.3)
7a				129.1 (117.8)	118.8 (117.8)	
7b				126.0 (123.4)	123.8 (123.4)	
8	132.3 (131.3)	130.7 (136.0)	131.4 (130.5)			124.6 (125.2)
8a				152.5 (138.5)	138.5 (138.5)	
9	118.3 (119.5)	120.3 (123.4)	110.7 (112.5)	118.0 (116.1)	115.9 (116.1)	126.2 (124.4)
9a	163.4 (161.0)		143.6 (145.0)			125.1 (123.8)
10		126.7 (127.2)		130.8 (131.4)	131.8 (131.4)	133.2 (133.3)
10a	141.0 (142.0)	123.3 (116.3)	129.5 (130.6)			141.1 (136.9)
10b		126.0 (134.2)				
11	128.9 (127.3)	135.3 (130.0)	119.3 (120.8)	120.9 (122.1)	121.8 (122.2)	
11a	120.1 (121.5)	120.0 (125.5)	122.4 (122.2)			162.0 (161.4)
12				131.9 (125.6)	126.2 (125.5)	
12a				135.1 (116.8)	117.4 (116.8)	
13				130.5 (93.2)	90.9 (93.3)	
13a				136.8 (137.3)	137.6 (137.3)	
13b				129.0 (120.4)	120.1 (120.4)	
*N*5-Me	31.2 (29.1)	41.9 (43.1)		28.4 (36.2)	36.3 (36.2)	
*i*-Pr-CH						39.2 (39.8)
C=O			195.6 (195.6)			
1′	133.8 (134.0)	129.4 (128.9)		126.6 (127.8)	127.7 (127.8)	114.3 (112.6)
2′		127.2 (127.3)		115.5 (119.2)	117.7 (119.2)	128.5 (121.4)
3′	140.7 (139.9)	128.5 (129.2)		127.3 (128.3)	128.7 (128.4)	120.8 (128.6)
4′	113.3 (115.0)	131.1 (129.1)		109.5 (113.0)	112.1 (113.1)	124.3 (124.4)
4a′	129.6 (128.7)	144.1 (145.9)		140.3 (140.9)	139.8 (140.9)	118.9 (117.7)
4b′	121.3 (121.4)					133.5 (132.9)
5′	121.1 (122.2)					
5a′		145.8 (147.4)		118.9 (121.2)	121.1 (121.2)	140.8 (141.4)
5b′		124.7 (123.8)		116.1 (117.5)	118.6 (117.5)	
6′	119.6 (121.0)	124.1 (123.6)		117.5 (119.8)	120.0 (119.9)	116.0 (116.6)
7′	128.1 (128.6)	123.0 (123.7)		115.8 (118.3)	118.4 (118.4)	132.6 (132.2)
8′	111.7 (111.6)	131.7 (132.1)		119.9 (121.9)	122.1 (121.9)	121.2 (121.8)
8a’	142.8 (142.1)					
9′		114.2 (111.9)		108.4 (112.0)	111.5 (112.1)	127.0 (126.1)
9a′	137.1 (137.4)	144.0 (146.1)		133.3 (135.6)	134.0 (135.7)	125.4 (124.9)
10′						165.7 (167.1)
10a′		131.6 (135.3)		111.7 (117.6)	118.1 (117.6)	124.1 (130.0)
11′		117.5 (115.8)				
11a′		127.9 (128.5)		120.0 (124.0)	126.9 (124.0)	140.6 (141.4)
*N*5′-Me				24.7 (36.2)	35.5 (36.1)	36.5 (37.0)

*^a^* Experimental values are given in parenthesis.

## Data Availability

Data is contained within the article and Supplementary Materials.

## Supplementary Materials

The following supporting information can be downloaded at: https://www.mdpi.com/article/10.3390/ijms26052112/s1.
