# Supplementary material for: Computational NMR Study of Cryptolepis Alkaloids: Could the Structural Misassignment of Cryptospirolepine Have Been Avoided?
Source: Int J Mol Sci. 2025 Feb 27;26(5):2112. doi: 10.3390/ijms26052112 (PMC11900956; doi:10.3390/ijms26052112)
Supplement: Supplementary file 1 [file ijms-26-02112-s001.zip › ijms-3446970-supplementary.pdf]

SUPPORTING INFORMATION

Computational NMR Study of *Cryptolepis* Alkaloids: Could the Structural Misassignment of Cryptospirolepine Have Been Avoided?

Valentin A. Semenov,[1*] Gary E. Martin,[2*] and Leonid B. Krivdin[1]

[1] A.E. Favorsky Irkutsk Institute of Chemistry, Siberian Branch of the Russian Academy of Sciences, Favorsky St. 1, 664033 Irkutsk, Russia. E-mail: semenov@irioch.irk.ru
[2] Department of Chemistry and Biochemistry, Seton Hall University 400 South Orange Ave., South Orange, NJ 07079 USA. E-mail: gemartin1@shu.edu

TABLE OF CONTENTS

Cartesian coordinates (Angstroms) of compounds 1-13, optimized at the M06-2X/cc-pVTZ//aug-cc-pVTZ level in the IEF-PCM media. S3

Compound: **1**. Solvent: Chloroform. S3

Compound: **2**. Solvent: DiMethylSulfoxide. S4

Compound: **3**. Solvent: Chloroform. S5

Compound: **4**. Solvent: DiMethylSulfoxide. S6

Compound: **5a**. Solvent: DiMethylSulfoxide. S7

Compound: **5b**. Solvent: Chloroform. S8

Compound: **6**. Solvent: DiMethylSulfoxide. S9

Compound: **7**. Solvent: DiMethylSulfoxide. S10

Compound: **8**. Solvent: Dimethylsulfoxide. S12

Compound: **9**. Solvent: DiMethylSulfoxide. S13

Compound: **10**. Solvent: Methanol. S15

Compound: **11**. Solvent: DiMethylSulfoxide. S17

Compound: **12a**. Solvent: DiMethylSulfoxide. S19

Compound: **12b**. Solvent: DiMethylSulfoxide. S21

Compound: **13**. Solvent: DiMethylSulfoxide. S23

Figure S1. Calculated and experimental NMR chemical shifts values in cryptolepinone 5(*N*)-oxide (**6**). S25

Table S1. Calculated ^{1}H NMR chemical shifts of **1-13**, ppm. S26

Table S2. Calculated ^{13}C NMR chemical shifts of **1-13**, ppm. S27

Cartesian coordinates (Angstroms) of compounds 1-13, optimized at the M06-2X/cc-pVTZ//aug-cc-pVTZ level in the IEF-PCM media.

Compound: **1**. Solvent: Chloroform.

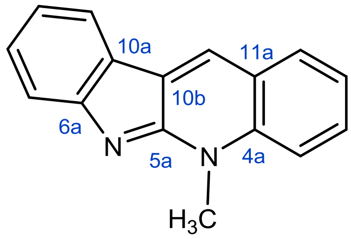

E^0: -725.8731226 a.u.

C	-0.544996000	-0.583352000	0.000266000
C	-0.266124000	0.850699000	0.000118000
N	1.021844000	1.290352000	0.000393000
C	2.071716000	0.392392000	-0.000038000
C	1.816083000	-0.999957000	0.000333000
C	0.467008000	-1.474461000	0.000678000
C	2.901257000	-1.891693000	0.000237000
C	4.197956000	-1.438950000	-0.000344000
C	4.439963000	-0.061140000	-0.000803000
C	3.401664000	0.843338000	-0.000576000
C	-1.990110000	-0.642019000	0.000315000
C	-2.402305000	0.709898000	-0.000109000
N	-1.331346000	1.608659000	0.000031000
C	-2.916976000	-1.675727000	0.000033000
C	-4.265857000	-1.353040000	-0.000296000
C	-4.674249000	-0.015923000	-0.000410000
C	-3.757054000	1.024780000	-0.000428000
C	1.274946000	2.726920000	0.000662000
H	0.289623000	-2.543328000	0.001375000
H	2.688382000	-2.953079000	0.000599000
H	5.022854000	-2.136642000	-0.000456000
H	5.456821000	0.306701000	-0.001396000
H	3.618710000	1.899954000	-0.000698000
H	-2.597329000	-2.710093000	0.000027000
H	-5.008482000	-2.138613000	-0.000462000
H	-5.732121000	0.211601000	-0.000615000
H	-4.078394000	2.057361000	-0.000659000
H	1.836234000	3.007690000	0.890264000
H	1.837946000	3.007154000	-0.888175000
H	0.314737000	3.227634000	-0.000591000

Compound: **2**. Solvent: DiMethylSulfoxide.

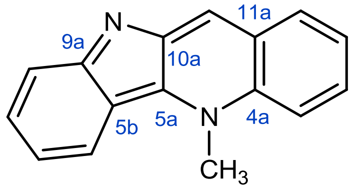

E^0: -725.8551387 a.u.

C	-0.243939000	-1.318304000	0.000074000
C	-0.435732000	0.118342000	0.000046000
N	0.606598000	0.972101000	0.000200000
C	1.901247000	0.502478000	0.000003000
C	2.121074000	-0.904853000	0.000176000
C	1.038014000	-1.803187000	0.000282000
C	3.454128000	-1.388267000	0.000177000
C	4.518257000	-0.536634000	-0.000184000
C	4.290303000	0.853917000	-0.000541000
C	3.021061000	1.365430000	-0.000491000
N	-1.420117000	-1.990835000	0.000036000
C	-2.359352000	-1.023337000	-0.000026000
C	-1.835189000	0.319575000	-0.000027000
C	0.276586000	2.397680000	0.000902000
H	1.227849000	-2.869300000	0.000502000
H	3.601407000	-2.460297000	0.000367000
H	5.528710000	-0.919314000	-0.000202000
H	5.129554000	1.535233000	-0.000955000
H	2.903732000	2.435260000	-0.001023000
H	-0.310050000	2.619646000	0.889683000
H	1.164481000	3.009006000	0.002461000
H	-0.308183000	2.621192000	-0.888738000
C	-3.760820000	-1.217411000	-0.000117000
C	-4.583584000	-0.124505000	-0.000224000
C	-4.069434000	1.197409000	-0.000286000
C	-2.719074000	1.424309000	-0.000199000
H	-4.156712000	-2.224194000	-0.000098000
H	-5.656688000	-0.265428000	-0.000289000
H	-4.755101000	2.032768000	-0.000422000
H	-2.355637000	2.440712000	-0.000327000

Compound: **3**. Solvent: Chloroform.

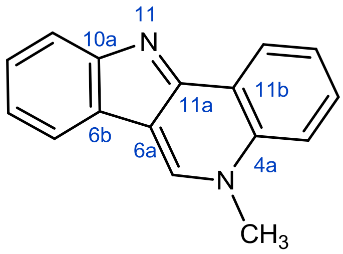

E^0: - 725.8693054 a.u.

C	0.623085000	0.686682000	-0.000910000
C	-0.198408000	1.770642000	-0.000206000
N	-1.542716000	1.643995000	-0.000032000
C	-2.150149000	0.389246000	-0.000052000
C	-1.362666000	-0.781179000	-0.000416000
C	0.068415000	-0.642990000	-0.000482000
C	-1.991879000	-2.033125000	-0.000499000
C	-3.362083000	-2.133982000	-0.000206000
C	-4.139258000	-0.970599000	0.000192000
C	-3.550031000	0.273447000	0.000302000
C	-2.373000000	2.844770000	0.000911000
N	0.975396000	-1.593130000	0.000262000
H	-1.361168000	-2.911419000	-0.000714000
H	-3.839194000	-3.103727000	-0.000342000
H	-5.218062000	-1.042340000	0.000508000
H	-4.174754000	1.152650000	0.000497000
H	-3.002737000	2.870676000	-0.886677000
H	-3.000599000	2.870794000	0.889948000
H	-1.724247000	3.713676000	0.000030000
C	2.049619000	0.485511000	-0.000464000
C	2.196281000	-0.924631000	0.000297000
C	3.468830000	-1.495947000	0.000904000
C	4.568641000	-0.656295000	0.000722000
C	4.422497000	0.737643000	-0.000174000
C	3.165442000	1.317751000	-0.000835000
H	0.173604000	2.785761000	-0.000136000
H	3.581270000	-2.571826000	0.001468000
H	5.563425000	-1.081838000	0.001181000
H	5.303543000	1.364697000	-0.000355000
H	3.058139000	2.395173000	-0.001530000

Compound: **4**. Solvent: DiMethylSulfoxide.

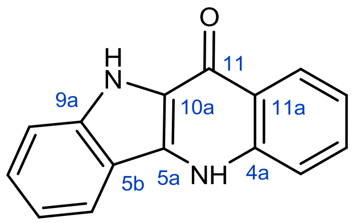

E^0: -761.8115464 a.u.

C	-1.822837000	-0.897927000	-0.000143000
C	-2.133067000	0.477599000	-0.000166000
C	-3.482992000	0.864443000	0.000047000
C	-4.490968000	-0.066896000	0.000243000
C	-4.168681000	-1.433948000	0.000241000
C	-2.860335000	-1.847285000	0.000039000
C	-1.085172000	1.509817000	-0.000550000
C	0.233250000	0.953200000	-0.000106000
C	0.492217000	-0.403886000	-0.000081000
N	-0.517106000	-1.315322000	-0.000302000
O	-1.313576000	2.722550000	0.000244000
N	1.430121000	1.637647000	-0.000265000
C	2.454574000	0.734447000	-0.000020000
C	1.913262000	-0.574892000	-0.000111000
C	3.836299000	0.945431000	0.000189000
C	4.651441000	-0.165575000	0.000259000
C	4.126269000	-1.472161000	0.000102000
C	2.765960000	-1.685551000	-0.000113000
H	-3.697397000	1.924463000	0.000009000
H	-5.525768000	0.244189000	0.000387000
H	-4.958299000	-2.172836000	0.000393000
H	-2.611135000	-2.900353000	0.000015000
H	-0.314622000	-2.302467000	0.000666000
H	1.510207000	2.640240000	0.000945000
H	4.245356000	1.945962000	0.000280000
H	5.724538000	-0.031184000	0.000432000
H	4.803140000	-2.314626000	0.000131000
H	2.366158000	-2.690957000	-0.000237000

Compound: **5a**. Solvent: DiMethylSulfoxide.

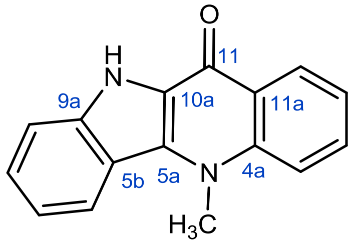

E^0: -801.0754181 a.u.

C	-1.824225000	-0.723456000	0.011519000
C	-2.114857000	0.661162000	0.008720000
C	-3.449772000	1.086105000	-0.063441000
C	-4.484244000	0.188350000	-0.142461000
C	-4.192304000	-1.181834000	-0.162636000
C	-2.896914000	-1.633929000	-0.089039000
C	-1.059000000	1.676102000	0.070489000
C	0.245752000	1.098243000	0.070225000
C	0.494305000	-0.262876000	0.063660000
N	-0.524603000	-1.176235000	0.099179000
O	-1.264148000	2.893829000	0.097265000
N	1.437566000	1.782580000	0.055209000
C	2.461046000	0.884435000	0.014534000
C	1.926061000	-0.431147000	0.010217000
C	3.836680000	1.125261000	-0.039784000
C	4.674973000	0.037164000	-0.117618000
C	4.168041000	-1.274355000	-0.153964000
C	2.814019000	-1.516148000	-0.095044000
H	-3.629074000	2.152538000	-0.057593000
H	-5.508171000	0.528677000	-0.199543000
H	-4.994302000	-1.902546000	-0.247155000
H	-2.708556000	-2.694684000	-0.137179000
C	-0.228554000	-2.587131000	0.310481000
H	1.512342000	2.785663000	0.037386000
H	4.219500000	2.136185000	-0.028805000
H	5.744156000	0.192740000	-0.163065000
H	4.853698000	-2.105614000	-0.236830000
H	2.457785000	-2.533233000	-0.150931000
H	0.700028000	-2.664863000	0.863605000
H	-1.006938000	-3.036889000	0.918082000
H	-0.144062000	-3.128689000	-0.631943000

Compound: **5b**. Solvent: Chloroform.

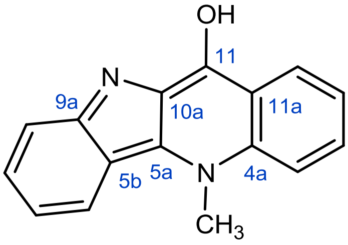

E^0: -801.0472939 a.u.

C	-1.802447000	-0.751716000	0.010994000
C	-2.077820000	0.643140000	0.006135000
C	-3.417851000	1.089663000	-0.037159000
C	-4.451999000	0.199921000	-0.090056000
C	-4.176454000	-1.180300000	-0.111985000
C	-2.891023000	-1.649505000	-0.063673000
C	-1.004488000	1.563533000	0.040180000
C	0.287925000	1.086737000	0.038449000
C	0.523654000	-0.319515000	0.034965000
N	-0.508205000	-1.203274000	0.073601000
O	-1.247021000	2.874459000	0.056853000
N	1.418520000	1.831304000	0.036404000
C	2.403749000	0.909936000	0.015189000
C	1.933558000	-0.454326000	0.005336000
C	3.794736000	1.164261000	-0.012718000
C	4.662321000	0.108967000	-0.069746000
C	4.201678000	-1.231855000	-0.111752000
C	2.863059000	-1.516763000	-0.078752000
H	-3.597709000	2.155229000	-0.032336000
H	-5.473960000	0.548397000	-0.126122000
H	-4.991222000	-1.888056000	-0.177304000
H	-2.719046000	-2.712671000	-0.111010000
C	-0.211309000	-2.625518000	0.217910000
H	4.146303000	2.187137000	0.003796000
H	5.728182000	0.294902000	-0.094932000
H	4.920351000	-2.036415000	-0.179684000
H	2.542552000	-2.546792000	-0.138718000
H	0.727712000	-2.719364000	0.749928000
H	-0.980692000	-3.104997000	0.812290000
H	-0.136978000	-3.111110000	-0.754599000
H	-0.385263000	3.321891000	0.063940000

Compound: **6**. Solvent: DiMethylSulfoxide.

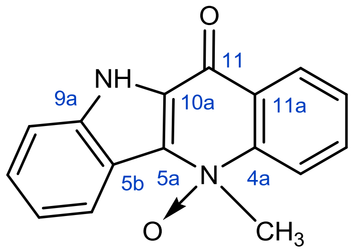

E^0: -876.1444639 a.u.

C	-1.840871000	-0.607432000	-0.040664000
C	-2.104655000	0.759453000	0.013191000
C	-3.425154000	1.193277000	-0.101996000
C	-4.453201000	0.285708000	-0.264163000
C	-4.166750000	-1.074646000	-0.328482000
C	-2.861237000	-1.523437000	-0.218676000
C	-1.023956000	1.774170000	0.118431000
C	0.300138000	1.189593000	0.063351000
C	0.550481000	-0.151314000	0.023575000
N	-0.470675000	-1.177424000	0.124058000
O	-1.226814000	2.969121000	0.202597000
N	1.485413000	1.867844000	0.018307000
C	2.507590000	0.960779000	-0.052431000
C	1.954928000	-0.343882000	-0.048040000
C	3.887667000	1.171783000	-0.126553000
C	4.693734000	0.058842000	-0.189225000
C	4.158680000	-1.246915000	-0.186624000
C	2.802984000	-1.461094000	-0.121312000
H	-3.614257000	2.257164000	-0.069242000
H	-5.473281000	0.630843000	-0.353090000
H	-4.964065000	-1.790498000	-0.470841000
H	-2.613945000	-2.571278000	-0.294385000
C	-0.390850000	-1.770820000	1.509336000
H	4.298300000	2.171450000	-0.134563000
H	5.765952000	0.186467000	-0.246251000
H	4.832505000	-2.090132000	-0.244684000
H	2.380915000	-2.455168000	-0.142962000
H	-0.605820000	-1.005380000	2.251653000
H	-1.116115000	-2.575874000	1.546524000
H	0.616895000	-2.156987000	1.620389000
O	-0.237550000	-2.167832000	-0.798374000
H	1.567497000	2.871752000	0.008794000

Compound: **7**. Solvent: DiMethylSulfoxide.

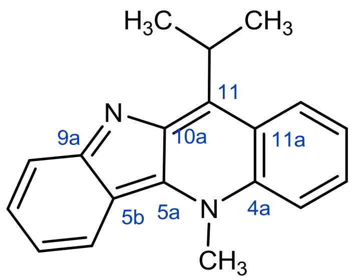

E^0: -843.6917545 a.u.

C	1.602548000	-1.319887000	0.031933000
C	2.006737000	0.047933000	0.001819000
C	3.402594000	0.300634000	-0.097275000
C	4.321944000	-0.705596000	-0.169669000
C	3.897751000	-2.046036000	-0.153322000
C	2.568541000	-2.347582000	-0.055324000
C	1.043137000	1.098802000	0.056685000
C	-0.292777000	0.731925000	0.031452000
C	-0.660366000	-0.667850000	0.050217000
N	0.270615000	-1.635961000	0.126166000
C	1.434481000	2.557771000	0.105901000
N	-1.392381000	1.532463000	-0.000491000
C	-2.437960000	0.683659000	-0.021301000
C	-2.074273000	-0.707026000	0.006061000
C	-3.806002000	1.040208000	-0.083673000
C	-4.749184000	0.050498000	-0.140137000
C	-4.389851000	-1.321163000	-0.149728000
C	-3.075397000	-1.701691000	-0.082348000
H	3.753339000	1.318902000	-0.134825000
H	5.373671000	-0.471638000	-0.253094000
H	4.620818000	-2.845549000	-0.232963000
H	2.262537000	-3.380601000	-0.081805000
C	-0.165317000	-3.015799000	0.343187000
H	-4.083284000	2.085987000	-0.094080000
H	-5.797520000	0.315122000	-0.190882000
H	-5.164043000	-2.072008000	-0.220424000
H	-2.830648000	-2.752991000	-0.119809000
H	-0.311389000	-3.530953000	-0.605054000
H	0.567833000	-3.541820000	0.942338000
H	-1.094468000	-2.987712000	0.898735000
C	1.062376000	3.261825000	-1.203932000
C	0.824773000	3.272418000	1.316733000
H	2.511753000	2.628894000	0.218299000
H	1.392778000	4.300402000	-1.170023000

H	1.540445000	2.778531000	-2.056579000
H	-0.015864000	3.240157000	-1.349188000
H	1.209411000	4.291692000	1.363898000
H	-0.258459000	3.304777000	1.238101000
H	1.092910000	2.765039000	2.243946000

Compound: **8**. Solvent: Dimethylsulfoxide.

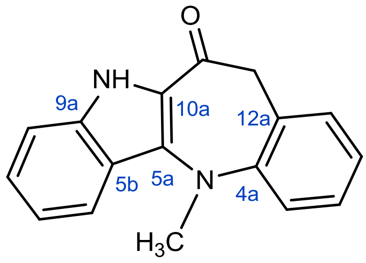

E^0: -840.3293153 a.u.

C	-0.667327000	2.172011000	0.344941000
C	0.421942000	1.256052000	0.100173000
C	0.534521000	-0.099162000	0.371137000
N	-0.500932000	-0.941679000	0.759771000
N	1.638136000	1.738107000	-0.339737000
C	2.550645000	0.738406000	-0.324003000
C	1.911705000	-0.452491000	0.110821000
C	3.905152000	0.778503000	-0.676826000
C	4.619855000	-0.391734000	-0.600248000
C	4.009479000	-1.591701000	-0.185048000
C	2.680449000	-1.633317000	0.165595000
C	-0.157632000	-2.259778000	1.271410000
H	4.362378000	1.704138000	-0.997950000
H	5.668524000	-0.394314000	-0.864554000
H	4.598295000	-2.497114000	-0.141548000
H	2.251921000	-2.572187000	0.476007000
H	0.662358000	-2.160736000	1.977997000
H	-1.019943000	-2.661980000	1.797341000
H	0.130276000	-2.963543000	0.486727000
C	-1.872507000	1.580021000	1.029793000
C	-1.785691000	-0.790156000	0.181842000
C	-2.453846000	0.437620000	0.242580000
O	-0.585729000	3.355704000	0.053521000
C	-3.693314000	0.566389000	-0.376210000
C	-4.295957000	-0.507689000	-1.011775000
C	-3.650616000	-1.736365000	-1.027624000
C	-2.404844000	-1.877108000	-0.437747000
H	1.795189000	2.705813000	-0.567349000
H	-2.607545000	2.367112000	1.175241000
H	-1.548391000	1.206210000	2.006381000
H	-4.202189000	1.520572000	-0.326150000
H	-5.265752000	-0.390829000	-1.474640000
H	-4.109054000	-2.587986000	-1.511329000
H	-1.902751000	-2.832789000	-0.477443000

Compound: **9**. Solvent: DiMethylSulfoxide.

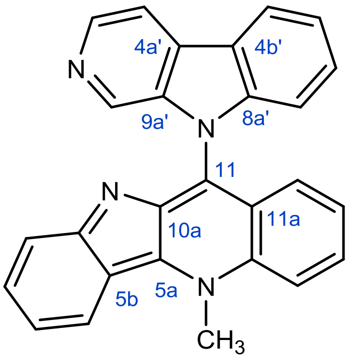

E^0: -1257.7416774 a.u.

C	-3.452736000	-0.531110000	0.644789000
C	-3.148391000	-1.412478000	-0.458805000
C	-1.841444000	-1.098006000	-0.873553000
N	-1.350938000	-0.056894000	-0.094380000
C	-2.321470000	0.278796000	0.838779000
C	-4.540775000	-0.346873000	1.490524000
C	-4.429991000	0.620838000	2.473350000
N	-3.341305000	1.383366000	2.661980000
C	-2.296473000	1.220533000	1.861059000
C	-3.862293000	-2.413156000	-1.117914000
C	-3.259797000	-3.073707000	-2.171023000
C	-1.956690000	-2.743799000	-2.571979000
C	-1.228774000	-1.756242000	-1.935376000
H	-5.442674000	-0.935246000	1.396782000
H	-5.253572000	0.797122000	3.153552000
H	-1.426784000	1.844387000	2.033747000
H	-4.868594000	-2.663638000	-0.809565000
H	-3.794782000	-3.853097000	-2.694975000
H	-1.508591000	-3.276776000	-3.399492000
H	-0.221766000	-1.508580000	-2.239311000
C	-0.048240000	0.474930000	-0.162066000
C	0.157236000	1.843048000	-0.468473000
C	1.477588000	2.367783000	-0.514806000
N	2.551624000	1.529233000	-0.323728000
C	2.366058000	0.241171000	0.008459000
C	1.037383000	-0.337829000	0.084819000
C	-0.924042000	2.714156000	-0.755141000
C	-0.717078000	4.037596000	-1.013338000
C	0.590462000	4.559994000	-0.989388000
C	1.664724000	3.747626000	-0.753002000
N	1.061530000	-1.642772000	0.419183000
C	2.376914000	-1.922796000	0.577313000

C	3.255065000	-0.807552000	0.350032000
C	2.922562000	-3.169301000	0.953744000
C	4.278206000	-3.278785000	1.113915000
C	5.142701000	-2.173201000	0.922169000
C	4.646880000	-0.951212000	0.550714000
C	3.922324000	2.011812000	-0.508133000
H	-1.924804000	2.307308000	-0.769793000
H	-1.553739000	4.686186000	-1.229556000
H	0.752404000	5.614672000	-1.160885000
H	2.651828000	4.178916000	-0.725777000
H	2.263336000	-4.011048000	1.117260000
H	4.704963000	-4.229603000	1.405568000
H	6.204506000	-2.296158000	1.080991000
H	5.327314000	-0.120472000	0.438753000
H	3.939893000	2.785336000	-1.265108000
H	4.323293000	2.393794000	0.429113000
H	4.521937000	1.182965000	-0.862694000

Compound: **10**. Solvent: Methanol.

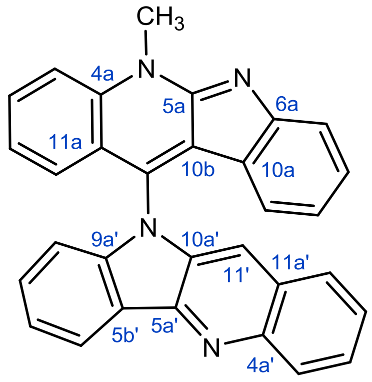

E^0: -1411.2805008 a.u.

C	1.641417000	-2.281318000	1.167470000
C	2.312151000	-1.323874000	0.307934000
C	1.291204000	-0.482575000	-0.239532000
N	0.062975000	-0.904337000	0.249950000
C	0.270045000	-1.988762000	1.104563000
C	2.097719000	-3.321094000	1.969648000
C	1.173753000	-4.050730000	2.697348000
C	-0.189535000	-3.741930000	2.629037000
C	-0.665104000	-2.708963000	1.836875000
N	3.587352000	-1.199499000	0.045168000
C	3.940071000	-0.197617000	-0.802264000
C	2.988997000	0.686942000	-1.392022000
C	1.617942000	0.529116000	-1.093057000
H	3.154714000	-3.544899000	2.019061000
H	1.503662000	-4.863885000	3.328006000
H	-1.719390000	-2.473094000	1.791537000
C	5.314203000	-0.028352000	-1.108719000
C	3.451206000	1.706508000	-2.262695000
H	0.878232000	1.192764000	-1.522649000
C	-1.177191000	-0.307300000	-0.055991000
C	-2.163047000	-1.046033000	-0.798952000
C	-3.399685000	-0.428754000	-1.107292000
N	-3.648393000	0.875016000	-0.725345000
C	-2.713366000	1.586268000	-0.044367000
C	-1.439549000	0.968491000	0.312019000
C	-1.921573000	-2.353270000	-1.256576000
C	-2.873019000	-3.046242000	-1.963023000
C	-4.103487000	-2.439786000	-2.237172000
C	-4.365582000	-1.154171000	-1.824588000
C	-0.752991000	2.001232000	1.053216000
C	-1.652091000	3.094785000	1.043633000
N	-2.843708000	2.819994000	0.370250000

C	-1.314338000	4.284301000	1.679861000
C	-4.920452000	1.513956000	-1.051734000
H	-0.964369000	-2.809450000	-1.047398000
H	-2.671713000	-4.050151000	-2.307323000
H	-5.317066000	-0.705444000	-2.060951000
C	-0.089247000	4.360652000	2.326027000
H	-5.055112000	1.545873000	-2.131187000
H	-5.741588000	0.962326000	-0.598275000
H	-4.893231000	2.521152000	-0.655840000
C	0.468432000	2.087427000	1.714774000
C	0.792833000	3.276529000	2.350885000
H	-1.998705000	5.121635000	1.671192000
H	0.188788000	5.278366000	2.827199000
H	1.735610000	3.366173000	2.871914000
H	1.152351000	1.250311000	1.744275000
H	-0.892770000	-4.322083000	3.210732000
C	4.781423000	1.841843000	-2.536125000
C	5.724602000	0.963063000	-1.951768000
H	2.724661000	2.375510000	-2.706065000
H	5.121073000	2.623102000	-3.201971000
H	6.775359000	1.082213000	-2.177529000
H	6.017852000	-0.710438000	-0.650928000
H	-4.860386000	-2.980266000	-2.788440000

Compound: **11**. Solvent: DiMethylSulfoxide.

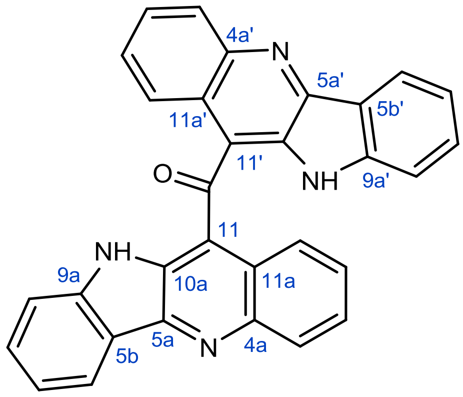

E^0: -1485.2744558 a.u.

C	1.384568000	-1.144827000	-0.276332000
C	2.460806000	-2.069537000	-0.139746000
C	3.797745000	-1.600440000	-0.304213000
N	4.100059000	-0.308589000	-0.602133000
C	3.101832000	0.520557000	-0.724479000
C	1.717669000	0.156574000	-0.563228000
C	2.268443000	-3.434269000	0.198860000
C	3.333278000	-4.277777000	0.346388000
C	4.654626000	-3.813635000	0.163936000
C	4.877177000	-2.505382000	-0.151048000
N	0.958735000	1.279727000	-0.767544000
C	1.784459000	2.358134000	-1.047758000
C	3.122972000	1.936490000	-1.033568000
C	1.441390000	3.678092000	-1.313606000
C	2.476468000	4.563755000	-1.565176000
C	3.816402000	4.156837000	-1.554806000
C	4.148485000	2.840110000	-1.289215000
H	1.265199000	-3.807386000	0.337160000
H	3.162500000	-5.313364000	0.606469000
H	5.484993000	-4.496069000	0.280487000
H	5.876351000	-2.115014000	-0.288275000
H	0.409693000	3.999895000	-1.323972000
H	2.241229000	5.598257000	-1.775396000
H	4.593396000	4.880361000	-1.756241000
H	5.178998000	2.511809000	-1.277934000
C	-0.041427000	-1.576983000	-0.190197000
O	-0.414744000	-2.564755000	-0.798317000
C	-1.031629000	-0.738604000	0.530522000
C	-2.315367000	-0.705216000	0.028199000
C	-3.280506000	0.219843000	0.570892000
N	-3.040328000	1.022622000	1.565228000

C	-1.807558000	0.948082000	2.142840000
C	-0.775442000	0.079410000	1.676846000
C	-4.487987000	0.059390000	-0.217063000
C	-4.207288000	-0.934708000	-1.167610000
N	-2.898365000	-1.372641000	-1.004584000
C	-5.740248000	0.660723000	-0.175933000
C	-6.696600000	0.254385000	-1.090820000
C	-6.404035000	-0.740363000	-2.030755000
C	-5.160940000	-1.351301000	-2.086281000
C	-1.571041000	1.780266000	3.263014000
C	-0.378059000	1.744549000	3.924567000
C	0.628864000	0.854276000	3.498060000
C	0.435912000	0.044827000	2.412376000
H	-2.457049000	-2.130905000	-1.498640000
H	-5.954237000	1.425980000	0.557805000
H	-7.678570000	0.705413000	-1.082082000
H	-7.167705000	-1.041712000	-2.734747000
H	-4.942262000	-2.118316000	-2.815467000
H	-2.375643000	2.431706000	3.575330000
H	-0.207960000	2.380547000	4.781892000
H	1.561588000	0.800774000	4.042403000
H	1.214004000	-0.648476000	2.130869000
H	-0.045090000	1.327155000	-0.717880000

Compound: **12a**. Solvent: DiMethylSulfoxide.

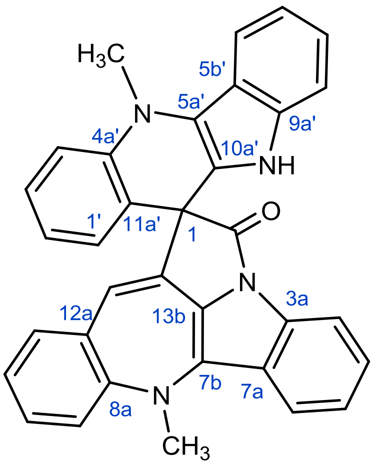

E^0: -1603.0497269 a.u.

C	-1.931344000	-3.065347000	3.491938000
C	-0.590836000	-2.752372000	3.304756000
C	-0.241929000	-1.921857000	2.253996000
C	-1.191482000	-1.401762000	1.379696000
C	-2.553560000	-1.720310000	1.570410000
C	-2.897485000	-2.554103000	2.646593000
N	-3.536819000	-1.211872000	0.741220000
C	-3.190700000	-0.164122000	-0.103753000
C	-1.890905000	0.163522000	-0.349461000
C	-0.717345000	-0.568020000	0.187222000
C	-4.013445000	0.776222000	-0.820994000
C	-3.110975000	1.632563000	-1.503043000
N	-1.835851000	1.237983000	-1.210750000
C	-5.390328000	1.027486000	-0.936505000
C	-5.819985000	2.076198000	-1.722212000
C	-4.906494000	2.899061000	-2.403296000
C	-3.547857000	2.692544000	-2.298546000
C	0.485209000	0.324326000	0.495794000
C	1.610834000	-0.285322000	-0.131218000
N	1.219773000	-1.325902000	-0.960554000
C	-0.135205000	-1.567857000	-0.881959000
O	-0.749259000	-2.407110000	-1.485385000
C	2.949668000	-0.141049000	-0.108655000
C	3.470569000	-1.155099000	-1.019397000
C	2.359029000	-1.886700000	-1.518355000
C	2.483080000	-2.946216000	-2.403135000
C	3.762293000	-3.284283000	-2.808960000
C	4.876506000	-2.575995000	-2.343972000
C	4.747521000	-1.521334000	-1.457996000
C	0.546400000	1.508120000	1.104356000

C	1.713595000	2.417976000	1.109730000
C	3.079493000	2.104870000	0.851679000
N	3.594260000	0.784598000	0.716168000
C	1.387696000	3.754715000	1.350311000
C	2.320006000	4.781269000	1.334740000
C	3.635796000	4.472259000	1.055210000
C	3.997098000	3.152972000	0.810498000
C	-4.886319000	-1.737393000	0.755524000
C	4.994219000	0.545940000	1.016127000
H	-2.233004000	-3.702471000	4.312303000
H	0.167941000	-3.145040000	3.966002000
H	0.800720000	-1.669575000	2.099859000
H	-3.935712000	-2.782617000	2.833972000
H	-0.994894000	1.719243000	-1.482711000
H	-6.115868000	0.424604000	-0.410541000
H	-6.879229000	2.273090000	-1.812592000
H	-5.275873000	3.712008000	-3.013243000
H	-2.838033000	3.329211000	-2.808919000
H	1.610691000	-3.475731000	-2.757033000
H	3.902317000	-4.104611000	-3.498741000
H	5.862040000	-2.859120000	-2.686749000
H	5.626500000	-0.992200000	-1.122139000
H	-0.362121000	1.922354000	1.527669000
H	0.352064000	3.988484000	1.561521000
H	2.015137000	5.797429000	1.540083000
H	4.393354000	5.243450000	1.029302000
H	5.032253000	2.935305000	0.596305000
H	-5.506634000	-1.287730000	1.535232000
H	-5.341535000	-1.553666000	-0.212793000
H	-4.860071000	-2.814700000	0.906631000
H	5.256654000	1.064062000	1.936344000
H	5.135183000	-0.521509000	1.171280000
H	5.675761000	0.877558000	0.226706000

Compound: **12b**. Solvent: DiMethylSulfoxide.

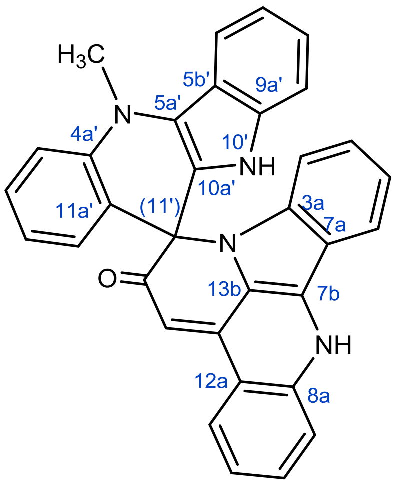

E^0: -1603.077695 a.u.

C	1.735130000	4.107378000	1.919592000
C	0.392634000	3.771523000	1.785166000
C	0.060268000	2.650486000	1.045619000
C	1.027251000	1.861322000	0.431544000
C	2.389238000	2.199097000	0.570567000
C	2.716968000	3.334953000	1.329954000
N	3.387819000	1.430687000	0.001084000
C	3.033009000	0.193432000	-0.523191000
C	1.734153000	-0.169792000	-0.708810000
C	0.551071000	0.677983000	-0.410111000
C	3.848204000	-0.937416000	-0.887900000
C	2.942108000	-1.945561000	-1.307407000
N	1.670828000	-1.456570000	-1.192159000
C	5.219822000	-1.237254000	-0.868581000
C	5.642843000	-2.483516000	-1.281358000
C	4.727253000	-3.457187000	-1.715691000
C	3.372304000	-3.203859000	-1.729496000
N	-0.461575000	-0.100875000	0.308999000
C	-1.791102000	-0.107335000	-0.027992000
C	-0.044241000	1.247512000	-1.750398000
O	0.762231000	1.799826000	-2.489723000
C	-2.532163000	-0.772231000	0.923644000
C	4.740332000	1.932869000	-0.138061000
H	2.024253000	4.972361000	2.501191000
H	-0.377458000	4.370702000	2.249045000
H	-0.982041000	2.373521000	0.930697000
H	3.753944000	3.595699000	1.479673000
H	5.945809000	-0.515224000	-0.524525000
H	6.698159000	-2.718247000	-1.266929000

H	5.091197000	-4.423554000	-2.036728000
H	2.660456000	-3.952539000	-2.049426000
H	5.346637000	1.762475000	0.754855000
H	5.209088000	1.442124000	-0.985488000
H	4.718009000	3.000799000	-0.347370000
C	-1.438543000	1.140515000	-2.039747000
C	-2.304263000	0.478913000	-1.196585000
C	-1.602791000	-1.228655000	1.928712000
H	0.822750000	-1.970412000	-1.363747000
H	-1.764540000	1.600616000	-2.960569000
C	-0.327569000	-0.760112000	1.508916000
C	0.826718000	-0.996842000	2.257462000
C	0.700467000	-1.736452000	3.414884000
C	-0.540507000	-2.246652000	3.824300000
C	-1.684955000	-2.004050000	3.095185000
C	-3.728227000	0.268624000	-1.362683000
C	-4.477616000	-0.425247000	-0.374573000
N	-3.887681000	-0.889959000	0.783958000
C	-4.393662000	0.734228000	-2.510529000
C	-5.735291000	0.527290000	-2.703563000
C	-6.462174000	-0.178002000	-1.736731000
C	-5.852044000	-0.645838000	-0.599451000
H	1.786558000	-0.619570000	1.934521000
H	1.579100000	-1.936089000	4.012706000
H	-0.599777000	-2.842750000	4.723819000
H	-2.620244000	-2.428740000	3.425861000
C	-4.691425000	-1.417551000	1.879208000
H	-6.433254000	-1.209626000	0.112522000
H	-7.516099000	-0.368694000	-1.885196000
H	-6.223337000	0.893296000	-3.595134000
H	-3.822183000	1.264293000	-3.258943000
H	-4.917118000	-2.474854000	1.740341000
H	-4.145653000	-1.276825000	2.803849000
H	-5.617047000	-0.855791000	1.958230000

Compound: **13**. Solvent: DiMethylSulfoxide.

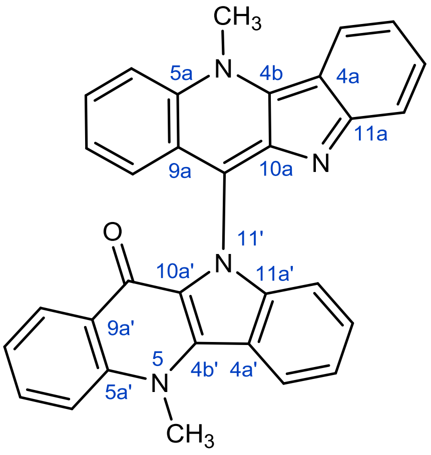

E^0: -1525.7221956 a.u.

N	0.391173000	0.488870000	0.515191000
C	1.625798000	0.090001000	0.013455000
C	2.576577000	1.045481000	0.329584000
O	1.053222000	-1.896569000	-1.121016000
C	1.897161000	-1.056790000	-0.798849000
C	1.905541000	2.109591000	1.036725000
C	0.552846000	1.708953000	1.128836000
C	-0.411812000	2.486047000	1.771992000
C	-0.011496000	3.695068000	2.294147000
C	1.316926000	4.137718000	2.175382000
C	2.271806000	3.363530000	1.555106000
C	3.297617000	-1.109751000	-1.238699000
C	4.243553000	-0.131037000	-0.864032000
N	3.885607000	0.914284000	-0.037201000
C	3.692052000	-2.165400000	-2.073493000
C	4.978032000	-2.265460000	-2.541918000
C	5.908276000	-1.280961000	-2.185653000
C	5.556185000	-0.234446000	-1.367596000
H	1.019509000	-1.408729000	2.044843000
H	-0.736611000	4.323431000	2.792819000
H	1.592413000	5.103770000	2.573841000
H	3.278250000	3.740861000	1.464108000
C	4.889078000	1.816488000	0.514526000
H	6.289881000	0.521884000	-1.139064000
H	6.919922000	-1.332322000	-2.564567000
H	5.269176000	-3.083652000	-3.184802000
H	2.939261000	-2.895797000	-2.335677000
C	-0.820719000	-0.233357000	0.479692000
C	-0.955931000	-1.451126000	1.189723000
C	-2.172188000	-2.179525000	1.120291000

N	-3.213980000	-1.695236000	0.361670000
C	-3.132214000	-0.486113000	-0.218748000
C	-1.902202000	0.282280000	-0.197934000
C	-4.046809000	0.285404000	-0.975926000
C	-3.272907000	1.436480000	-1.356176000
N	-1.999553000	1.431172000	-0.900683000
C	-2.303293000	-3.379606000	1.853217000
C	-1.263164000	-3.842058000	2.611599000
C	-0.048933000	-3.132472000	2.679804000
C	0.094994000	-1.965886000	1.986769000
C	-5.411522000	0.199461000	-1.333470000
C	-5.968619000	1.204899000	-2.078833000
C	-5.194923000	2.321123000	-2.483234000
C	-3.877611000	2.450015000	-2.132934000
C	-4.418748000	-2.498392000	0.145005000
H	5.817268000	1.276787000	0.668968000
H	4.548980000	2.158661000	1.484689000
H	5.068648000	2.670270000	-0.138930000
H	-3.232351000	-3.925808000	1.848212000
H	-1.384866000	-4.756551000	3.174747000
H	0.761175000	-3.506975000	3.288982000
H	-1.436895000	2.150903000	1.834779000
H	-6.031071000	-0.625942000	-1.015588000
H	-7.011796000	1.153756000	-2.356383000
H	-5.666711000	3.095070000	-3.074667000
H	-5.156171000	-2.303265000	0.921852000
H	-4.821717000	-2.240701000	-0.826578000
H	-4.159622000	-3.549696000	0.133061000
H	-3.295304000	3.311998000	-2.429492000

Figure S1. Calculated and experimental NMR chemical shifts values in cryptolepinone 5(*N*)-oxide (**6**). Black – experimental values, blue – computational assignments based on the performed calculations. ND means not determined.

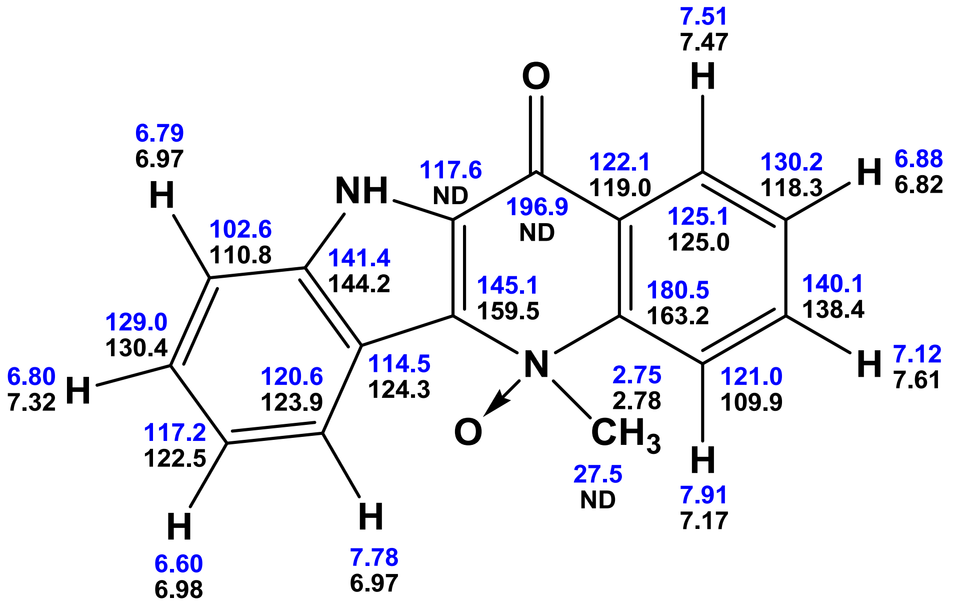

Table S1. Calculated ^{1}H NMR chemical shifts of **1-13**, ppm.

Nuclei	**1**	**2**	**3**	**4**	**5a**	**5b**	**6**	**7**	**8**	**9**	**10**	**11**	**12a**	**12b**	**13**
1	8.17	8.36	9.17	8.53	8.53	8.60	7.51	8.81	7.25	7.95	7.96	8.16			7.47
2	7.60	7.73	7.78	7.29	7.39	7.52	6.88	7.76	7.15	7.63	7.64	7.30			7.59
3	7.95	7.96	7.87	7.67	7.77	7.80	7.12	7.95	7.25	7.99	8.15	7.60			7.15
4	7.96	8.43	7.91	7.54	7.78	8.02	7.91	8.40	7.40	8.55	8.30	8.32	8.00	6.80	8.53
5													7.50	6.94	
6		8.55	8.50	8.02	8.34	8.27	7.78	8.51	8.28	8.58		8.44	7.55	7.12	8.41
7	7.76	7.18	8.15	7.22	7.28	6.95	6.60	7.08	7.03	7.17	7.87	7.29	8.09	8.28	7.87
8	7.66	7.70	7.39	7.52	7.54	7.43	6.80	7.62	7.31	7.62	7.67	7.50			7.47
9	7.32	7.76	7.61	7.50	7.55	7.66	6.79	7.73	7.25	7.46	6.96	7.08	6.86	7.85	7.88
10	8.24		7.96								6.76		7.43	7.84	
11	8.72	8.72											6.97	7.50	
12/12a									3.64				6.68	8.34	
12b									3.59						
13													5.86	6.16	
N-Me	4.26	4.86	4.12		4.34	4.38	2.75	4.72	3.98	4.99	5.17		3.66	4.34	5.01
i-Pr-CH								4.25							
i-Pr-Me								1.84							
1'										8.12	7.90		7.78	6.78	7.02
2'											7.77		7.19	6.61	7.44
3'										8.46	7.99		7.64	7.26	7.41
4'										8.20	8.51		7.37	7.13	8.54
5'										8.41					
6'										7.48	8.80		8.36	8.16	7.88
7'										7.54	7.79		7.42	7.17	7.78
8'										7.16	7.85		7.50	7.18	7.31
9'											7.42		7.60	7.16	8.23
11'											7.72				
N5'-Me													4.06	3.96	4.54

Table S2. Calculated ^{13}C NMR chemical shifts of **1-13**, ppm.

Nuclei	**1**	**2**	**3**	**4**	**5a**	**5b**	**6**	**7**	**8**	**9**	**10**	**11**	**12a**	**12b**	**13**
1	130.0	130.7	125.8	126.4	126.9	124.1	125.1	125.0	129.7	125.1	128.2	124.5	54.5	66.9	119.9
2	120.6	123.2	124.8	120.8	120.7	121.0	130.2	122.5	124.8	123.7	123.5	127.3	170.8	187.9	132.6
3	130.0	129.0	129.0	132.3	132.4	129.7	140.1	127.8	127.0	128.7	132.8	126.5			116.3
3a													130.9	140.2	
4	113.7	115.2	116.1	117.4	115.8	114.0	121.0	115.8	118.8	115.6	117.9	130.9	112.3	112.2	126.7
4a	135.9	131.0	135.0	138.7	141.0	134.3	180.5	131.7	149.4	132.4	138.0	143.7			115.9
4b															142.2
5													122.3	126.4	
5a	152.8	138.6		127.5	130.6	136.6	145.1	137.0	136.2	142.8	154.1	147.8			133.1
5b		115.3		116.1	118.2	115.2	114.5	113.3	120.9	114.0		121.3			
6		126.3	134.4	120.4	124.2	123.0	120.6	124.8	125.3	125.5		122.1	122.6	119.8	116.9
6a	155.4		118.0								155.0				
6b			125.9												
7	117.4	115.4	118.8	119.0	119.4	114.6	117.2	114.0	118.3	114.8	119.5	120.4	119.2	123.2	129.3
7a													129.1	118.8	
7b													126.0	123.8	
8	128.6	130.9	119.4	128.9	128.5	128.7	129.0	129.7	127.5	132.3	130.7	131.4			124.6
8a													152.5	138.5	
9	118.0	119.2	125.9	111.8	112.6	119.7	102.6	118.4	111.7	118.3	120.3	110.7	118.0	115.9	126.2
9a		160.5		137.9	138.0	158.5	141.4	157.7	135.3	163.4		143.6			125.1
10	120.7		119.4								126.7		130.8	131.8	133.2
10a	123.6	143.5	156.1	122.0	122.9	130.6	117.6	141.9	121.6	141.0	123.3	129.5			141.1
10b	128.0										126.0				
11	128.4	127.7		167.3	165.8	152.9	196.9	147.3	181.8	128.9	135.3	119.3	120.9	121.8	
11a	120.3	124.8	152.8	123.4	124.5	112.3	122.1	121.4		120.1	120.0	122.4			162.0
11b			122.2												
12									48.4				131.9	126.2	
12a									129.5				135.1	117.4	
13													130.5	90.9	
13a													136.8	137.6	
13b													129.0	120.1	
N5-Me	32.7	38.9	42.2		35.3	31.6	27.5	38.3	39.9	31.2	41.9		28.4	36.3	
i-Pr-CH								32.0							39.2
i-Pr-Me								20.4							
C=O												195.6			
1'										133.8	129.4		126.6	127.7	114.3
2'											127.2		115.5	117.7	128.5
3'										140.7	128.5		127.3	128.7	120.8
4'										113.3	131.1		109.5	112.1	124.3
4a'										129.6	144.1		140.3	139.8	118.9
4b'										121.3					133.5
5'										121.1					
5a'											145.8		118.9	121.1	140.8
5b'											124.7		116.1	118.6	
6'										119.6	124.1		117.5	120.0	116.0

7'										128.1	123.0		115.8	118.4	132.6
8'										111.7	131.7		119.9	122.1	121.2
8a'										142.8					
9'											114.2		108.4	111.5	127.0
9a'										137.1	144.0		133.3	134.0	125.4
10'															165.7
10a'											131.6		111.7	118.1	124.1
11'											117.5				
11a'											127.9		120.0	126.9	140.6
N5'-Me													24.7	35.5	36.5
